# Long-Term Efficacy and Safety of Pediatric Prolonged-Release Melatonin for Insomnia in Children with Autism Spectrum Disorder

**DOI:** 10.1089/cap.2018.0020

**Published:** 2018-12-10

**Authors:** Athanasios Maras, Carmen M. Schroder, Beth A. Malow, Robert L. Findling, John Breddy, Tali Nir, Shiri Shahmoon, Nava Zisapel, Paul Gringras

**Affiliations:** ^1^ Yulius Academy, Yulius Mental Health Organization, Barendrecht, The Netherlands.; ^2^ Strasbourg University Hospital Department of Child and Adolescent Psychiatry, Strasbourg, France.; ^3^ CNRS UPR 3212, Department of Psychiatry and Mental Health, Institute of Cellular and Integrative Neurosciences, Strasbourg, France.; ^4^ Sleep Division, Department of Neurology, Vanderbilt University Medical Center, Nashville, Tennessee.; ^5^ Department of Psychiatry and Behavioral Sciences, Kennedy Krieger Institute/Johns Hopkins University, Baltimore, Maryland.; ^6^ Pharmastat Consulting Ltd., Canterbury, United Kingdom.; ^7^ Neurim Pharmaceuticals Ltd., Tel Aviv, Israel.; ^8^ Children's Sleep Medicine, Evelina London Children's Hospital, Guy's and St. Thomas', London, United Kingdom.

**Keywords:** melatonin, insomnia, long-term, pediatric, autism, sleep disorders

## Abstract

***Objective:*** A recent double-blind randomized placebo-controlled study demonstrated 3-month efficacy and safety of a novel pediatric-appropriate prolonged-release melatonin (PedPRM) for insomnia in children and adolescents with autism spectrum disorder (ASD) and neurogenetic disorders (NGD) with/without attention-deficit/hyperactivity disorder comorbidity. Long-term efficacy and safety of PedPRM treatment was studied.

***Methods:*** A prospective, open-label efficacy and safety follow-up of nightly 2, 5, or 10 mg PedPRM in subjects who completed the 13-week double-blind trial (51 PedPRM; 44 placebo). Measures included caregiver-reported Sleep and Nap Diary, Composite Sleep Disturbance Index (CSDI), caregiver's Pittsburgh Sleep Quality Index (PSQI), Epworth Sleepiness Scale, and quality of life (WHO-5 Well-Being Index).

***Results:*** Ninety-five subjects (74.7% males; mean [standard deviation] age, 9 [4.24]; range, 2–17.5 years) received PedPRM (2/5 mg) according to the double-blind phase dose, for 39 weeks with optional dose adjustment (2, 5, or 10 mg/day) after the first 13 weeks. After 52 weeks of continuous treatment (PedPRM-randomized group) subjects slept (mean [SE]) 62.08 (21.5) minutes longer (*p* = 0.007); fell asleep 48.6 (10.2) minutes faster (*p* < 0.001); had 89.1 (25.5) minutes longer uninterrupted sleep episodes (*p* = 0.001); 0.41 (0.12) less nightly awakenings (>50% decrease; *p* = 0.001); and better sleep quality (*p* < 0.001) compared with baseline. The placebo-randomized group also improved with PedPRM. Altogether, by the end of 39-week follow-up, regardless of randomization assignment, 55/72 (76%) of completers achieved overall improvement of ≥1 hour in total sleep time (TST), sleep latency or both, over baseline, with no evidence of decreased efficacy. In parallel, CSDI child sleep disturbance and caregivers' satisfaction of their child's sleep patterns (*p* < 0.001 for both), PSQI global (*p* < 0.001), and WHO-5 (*p* = 0.001) improved in statistically significant and clinically relevant manner (*n* = 72) compared with baseline. PedPRM was generally safe; most frequent treatment-related adverse events were fatigue (5.3%) and mood swings (3.2% of patients).

***Conclusion:*** PedPRM, an easily swallowed formulation shown to be efficacious versus placebo, is an efficacious and safe option for long-term treatment (up to 52 weeks reported here) of children with ASD and NGD who suffer from insomnia and subsequently improves caregivers' quality of life.

## Introduction

Autism Spectrum Disorder (ASD) is characterized by persistent difficulties in social communication, as well as restricted interests and repetitive behaviors. The deficits result in functional limitations in effective communication, social participation, social relationships, academic achievement, or occupational performance, individually or in combination (APA 2013). Between 30% and 50% of individuals with ASD manifest attention-deficit/hyperactivity disorder (ADHD) symptoms (particularly at preschool age; Leitner 2014). Children with neuropsychiatric disorders (e.g., ASD and ADHD), neurogenetic disorders (NGD, e.g., Rett's disorder, tuberous sclerosis, Smith-Magenis Syndrome [SMS], and Angelman syndrome), and chronic neurologic disorders, such as epilepsy and Tourette's disorder, commonly exhibit chronic sleep disturbances (Mindell et al. 2006; Johnson and Malow 2008; Krakowiak et al. 2008; Hollway and Aman 2011; Kotagal and Broomall 2012; APA 2013; Elrod and Hood 2015). Compared with their age-matched typically developing peers, children with ASD are more likely to experience chronic sleep problems, with a prevalence of 50%–80% (Cuomo et al. 2017). The most prevalent complaints are difficulty falling asleep (∼40%) or maintaining sleep (∼35%; Taira et al. 1998; Krakowiak et al. 2008). Sleep problem severity is similar across ADHD and non-ADHD ASD subgroups (Taira et al. 1998; Krakowiak et al. 2008).

Sleep problems in children and adolescents with ASD are particularly challenging to their families and have been associated with increased maternal distress and parental sleep disruption as well as poor caregiver's quality of life (Doo and Wing 2006; Kuhlthau et al. 2010; Devnani and Hegde 2015).

Clinical guidelines recommend sleep hygiene and/or behavioral intervention as the first-line treatment (Howes et al. 2018). However many patients face limited access to behavioral intervention (e.g., limited caregiver time), and only around 25% respond to such therapy (Appleton et al. 2012). Patients with persistent problems will seek pharmacotherapy. However, there are no medications with regulatory approval for the treatment of chronic insomnia in children and adolescents (Mindell et al. 2006; Hollway and Aman 2011), and that is particularly problematic for children with ASD (Mindell et al. 2006; Johnson and Malow 2008; Efron et al. 2014). Consequently, physicians often prescribe off-label drugs, for example, antihistamines, alpha-adrenergic agonists (clonidine), antidepressants, and antipsychotics, for their sedative side effects without proven safety, efficacy, or dosing regimen in children (Mindell et al. 2006; Johnson and Malow 2008; Efron et al. 2014). Moreover, unregulated melatonin preparations or food supplements are used despite considerable concerns over the quality and potential safety hazards and lack of evidence on long-term efficacy and safety (Hollway and Aman 2011; Erland and Saxena 2017).

Melatonin treatment has shown promise in the treatment of insomnia in children with neurodevelopmental disabilities (Rossignol and Frye 2011; Cortesi et al. 2012; Gringras et al. 2012). A recent review of sleep-based interventions for children with ASD concluded that melatonin and behavioral interventions are the most effective of the current treatment options used for this condition (Cuomo et al. 2017). However, there is currently only one melatonin product with regulatory approval in the European Union and other countries (prolonged release melatonin for insomnia in patients aged 55 and over) and no melatonin products approved for use in children. Difficulty in swallowing may also limit the use of adult prolonged-release preparations in children (Schirm et al. 2003). Whereas periodic re-evaluation of the ongoing need of using melatonin in the treatment of sleep problems in children with ASD should be recommended as part of good clinical practice (GCP), data needed to underpin evidence-based clinical recommendations about useful duration dose and safety of melatonin treatment in such children are missing. Furthermore, no long-term well-controlled studies exist to support chronic use of such treatment in children and adolescents.

Pediatric prolonged-release melatonin (PedPRM; Neurim Pharmaceuticals) is a novel age-appropriate formulation (≤3 mm in diameter) under development for sleep disorders in children with neurodevelopmental disabilities who have difficulty swallowing. PedPRM is an oral solid dosage form of prolonged-release melatonin mini-tablets to be swallowed as a whole. PedPRM has been designed to gradually release melatonin, mimicking the physiological secretion profile of melatonin that produces sustained plasma levels of melatonin for up to 8–10 hours.

In a recent randomized, double-blind, placebo-controlled, parallel group multi-center (EU and United States) study, we investigated the effects of a new pediatric age-appropriate formulation of prolonged-release melatonin (PedPRM) for 13 weeks in children and adolescents (2–17.5 years old; *n* = 125) with ASD and NGD with or without ADHD comorbidity, who had not shown improvement after standard sleep behavioral intervention (Gringras et al. 2017).

Results of the 13-week double-blind phase of the study indicated that PedPRM (2/5 mg) was efficacious and safe compared with placebo for treatment of insomnia in children with ASD with/without ADHD and NGD with clinically meaningful improvements in total sleep time (TST), duration of uninterrupted sleep (longest sleep episode [LSE]), and sleep latency (SL) and without causing earlier wake-up time (Gringras et al. 2017).

A major aim of the study was to evaluate long-term safety of PedPRM treatment, thus providing clinicians with evidence-based long-term data. Here we report on the results of a prospective, 39-week, open-label, follow-up PedPRM treatment of the aforementioned study, resulting in up to 52 weeks (1 year) of continuous PedPRM treatment. The purpose of this follow-up was to describe long-term (up to 52 weeks) efficacy and safety of PedPRM at the optimal (2, 5, or 10 mg) daily dose and impact of the treatment on caregivers' sleep, daytime sleepiness, and quality of life.

## Methods

### Participants

Children and adolescents (2–17.5 years) with (1) physician-diagnosed ASD according to ICD-10/*Diagnostic and Statistical Manual of Mental Disorders, Fourth Edition* (DSM-4; American Psychiatric Association 1994)/*Diagnostic and Statistical Manual of Mental Disorders*, *Fifth Edition* (DSM-5; American Psychiatric Association 2013) criteria or (2) NGD who completed the double-blind phase of the study and were willing to continue into the open-label follow-up. The presence of comorbidities (e.g., enlarged tonsils and adenoids, or gastroesophageal reflux disease) was recorded and the child was allowed to be included in the study provided that the physician investigators considered such comorbidities not to be the cause for the sleep problem.

Exclusion criteria included other sleep disorders (e.g., moderate to severe sleep apnea), use of prohibited medication as detailed before (Gringras et al. 2017), melatonin within 2 weeks before screening, allergy to melatonin or lactose, or unresponsive to previous Circadin^®^ therapy participation in a clinical trial within the last 3 months before the study. Females not using contraceptives but were sexually active and pregnant and/or breastfeeding females were excluded.

### Study design

The study was carried out in 14 centers in the United States and 10 centers in Europe: United Kingdom, France, the Netherlands, and Finland. The 13-week, double-blind phase was conducted from December 2013 to May 2016. The 39-week, open-label follow-up took place from March 2014 to February 2017. The protocol and informed consent form were reviewed and approved by the institutional review boards of participating institutions. The trial complied with the principles of the Declaration of Helsinki (1989) and standards of GCP.

All participants and parents/legal guardians provided informed assent and consent, respectively, under procedures and local regulations of each country (ClinicalTrials.gov identifier: NCT01906866).

The study comprised 2-week, single-blind, placebo run-in (baseline), a 13-week, randomized double-blind efficacy and safety study of PedPRM (Neurim Pharmaceuticals Ltd.) or placebo treatment (2 mg, with optional dose escalation to 5 mg after 3 weeks if the patient did not improve from baseline by at least 1 hour in SL and/or TST), followed by a 91-week, open-label PedPRM treatment and 2-week, single-blind placebo period (withdrawal effects)—altogether 108 weeks (2.2 years) of study medication.

In the first 13 weeks of the follow-up, patients received PedPRM (2/5 mg) according to the double-blind phase dose. Accordingly, patients who received 2 mg placebo in the double-blind phase received 2 mg PedPRM, and those who were escalated to 5 mg placebo received 5 mg PedPRM. Once all subjects in the follow-up phase completed 39 weeks of follow-up (altogether 12 months of study medication), the 1-year data were summarized and are presented herein, while the patients continued in the study of additional 52 weeks of PedPRM treatment (ongoing).

Participants were instructed to take the study medication regularly after the evening meal, 30–60 minutes before bedtime. After the first 13 weeks of follow-up sleep variables were assessed, and if the patient did not improve from baseline by at least 1 hour in SL and/or TST in the double-blind or follow-up phases, the dose was escalated from 2 to 5 mg/day and from 5 to 10 mg/day. Optional decrease in dose was also allowed at all times during the study, based on the evaluation of excess drowsiness, behavioral changes, or ceasing to respond to study drug. Children then continued open-label on 2, 5, or 10 mg PedPRM for the remaining period, with efficacy assessment after 26 and 39 weeks of follow-up.

Treatment compliance was monitored in all subjects using a 3-monthly tablet count %adherence = 100*(number of tablets dispensed − number of tablets returned − number of tablets lost)/(Number of tablets per day × number of days since last visit).

Patients with significant adverse events were withdrawn from the study at the discretion of the investigator. All hypnotics or treatments used to induce sleep (including benzodiazepines, Z-drugs [benzodiazepine receptor agonists], herbal sleep preparations, antihistamines, alcohol) were not allowed during the study [Table S1, published online in Gringras et al. (2017)].

### Study endpoints

Sleep variables, reported by parent/caregiver, were assessed using a validated parent-reported Sleep and Nap Diary (SND). The SND was to be completed every morning by the parent/caregiver at home for 14 days before each visit. Child sleep variables included SND-recorded changes from baseline in mean TST, SL, number of awakenings (NOA), LSE (duration of uninterrupted sleep), and quality of sleep (QOS—“Can you describe the child's sleep quality last night as one of the following: Very bad, Bad, Fair, Good, Very good, Unknown”) over the 14 days and change from baseline in Composite Sleep Disturbance Index (CSDI) score.

Caregiver's measures included the Pittsburgh Sleep Quality Index (PSQI) score (Backhaus et al. 2002; Buysse et al. 2006), WHO-5 Well-Being index (Bech et al. 2003), Epworth Sleepiness Scale (ESS; Johns 1991), and CSDI-recorded satisfaction of child's sleep patterns. The PSQI comprises nine main questions relating to the patient's usual sleep habits during the previous 2 weeks. It addresses possible reasons for trouble in sleeping as well as daytime behavior. The caregiver is asked to give the most accurate reply for the majority of his/her own days and nights during this period. An algorithm is used to calculate seven component scores, and these are added to give a global PSQI score. The PSQI has been recommended as an essential measure for global sleep and insomnia symptoms in recent expert consensus recommendations for a standard set of research assessments in insomnia (Buysse et al. 2006). The WHO-5 Well-Being index covers positive mood, vitality, and general interests (Bech et al. 2003). A change of 10% and more in WHO-5 is considered clinically relevant (WHO 1998).

The ESS is a self-administered questionnaire with eight questions. Caregivers were asked to rate, on a four-point scale (0–3), their usual chances of dozing off or falling asleep while being engaged in eight different activities. The higher the ESS score, the higher that person's average sleep propensity in daily life, or their “daytime sleepiness” (Johns 1991).

Safety was monitored throughout the study, using standard clinical trials' methods and definitions (Treatment-Emergent Signs and Symptoms [TESS], AEs, vital signs, and physical examination). Child development was assessed using Tanner scale (pubertal stage), body mass index (BMI) percentiles (obesity), and Z scores (growth). Epilepsy and health status assessments were also assessed.

### Statistical methods

Efficacy analyses are presented for all participants who took at least one dose of study medication, satisfied all major entry criteria, and had valid assessments of mean TST at baseline and at least once during the double-blind phase. Within-participant changes from baseline were analyzed using paired *t*-tests. Missing data due to participant withdrawal or other reasons were not imputed.

## Results

### Study population

**FIG. 1.**
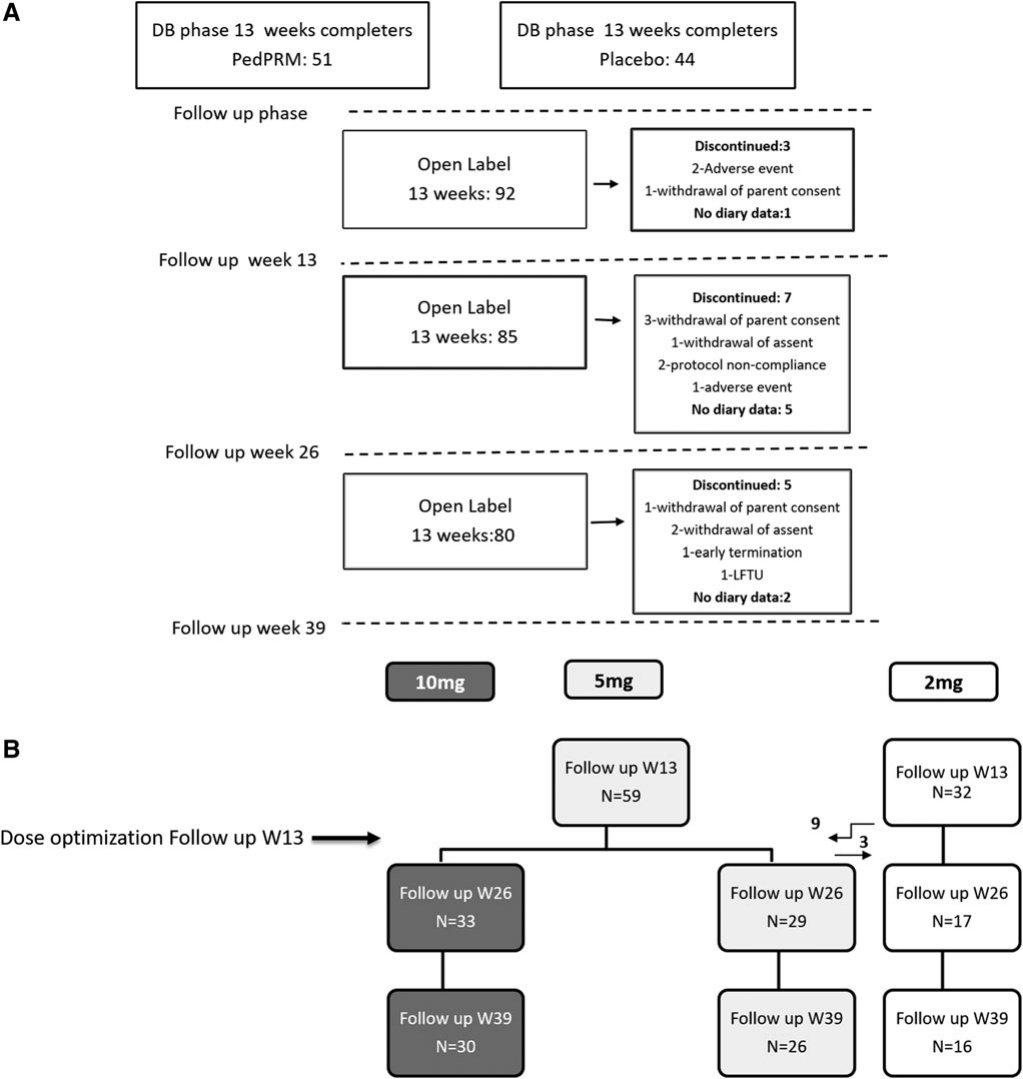
Overall study patient disposition (CONSORT diagram) **(A)** and dose breakdown for patients with SNDs **(B)**. The study comprised 9-month, open-label PedPRM treatment on 2 or 5 mg doses with optional dose adjustment after 13 weeks of the open-label phase. PedPRM, pediatric prolonged-release melatonin; SND, Sleep and Nap Diary.

One hundred twenty-five children (2–17.5 years; 96.8% ASD, 3.2% SMS) whose sleep failed to improve on behavioral intervention alone were randomized (1:1 ratio), double-blind, to receive PedPRM (2 mg escalated to 5 mg) or placebo for 13 weeks. A total of 95 participants who completed the 13-week double-blind phase entered the 39-week, open-label follow-up phase (Fig. 1). Mean (standard deviation [SD]) age was 9 (4.24); range was 2–17 years; 74.7% were males; and mean BMI was 19.8. Of the 95 patients in the follow-up phase, 51 had been assigned to PedPRM and 44 to placebo in the double-blind phase. Eighty subjects completed the 39-week follow-up (77 ASD, 3 SMS) and 15 discontinued; the most common reasons for discontinuation were withdrawal of parent consent (mainly because of personal reasons) and lost to follow-up (Fig. 1).

Of those who completed the 39-week follow-up, 25 (31%) had been diagnosed with comorbid ADHD (compared with 29% of randomized subjects at baseline).

Of those completing 39 weeks of follow-up, stimulant was reported by 15, clonidine by 6, and atomoxetine by 2. Patients remained on stable doses of these drugs throughout the follow-up.

Mixed effect model repeat measurement (MMRM) analyses including prior ADHD treatment, epilepsy, and prior clonidine use as factors did not reveal effects of the comedications on the response to PedPRM. Due to the small number of patients with each factor, no conclusions could be drawn.

Seventy-two of the 80 completers had valid SND data. Among the 23 patients who discontinued the study or did not have valid SND data, 15 (65.5%) attained clinically meaningful responses in TST and/or SL (improved on PedPRM by at least 45 minutes in TST or 15 minutes in SL or both, compared with baseline), based on their last valid SND data during the follow-up; this rate was similar to the respective number among patients assigned to PedPRM in the double-blind phase (68.9%; Gringras et al. 2017).

Treatment adherence was a mean 100% on average throughout the study. Principal investigators reported that there was no need to crush the mini-tablets and children were able to swallow the tablets, thus confirming previous findings on the acceptability and suitability of 3-mm-diameter minitablets for preschool-aged children (Thomson et al. 2009; Gringras et al. 2017).

### Efficacy

#### Sleep and Nap Diary

**FIG. 2.**
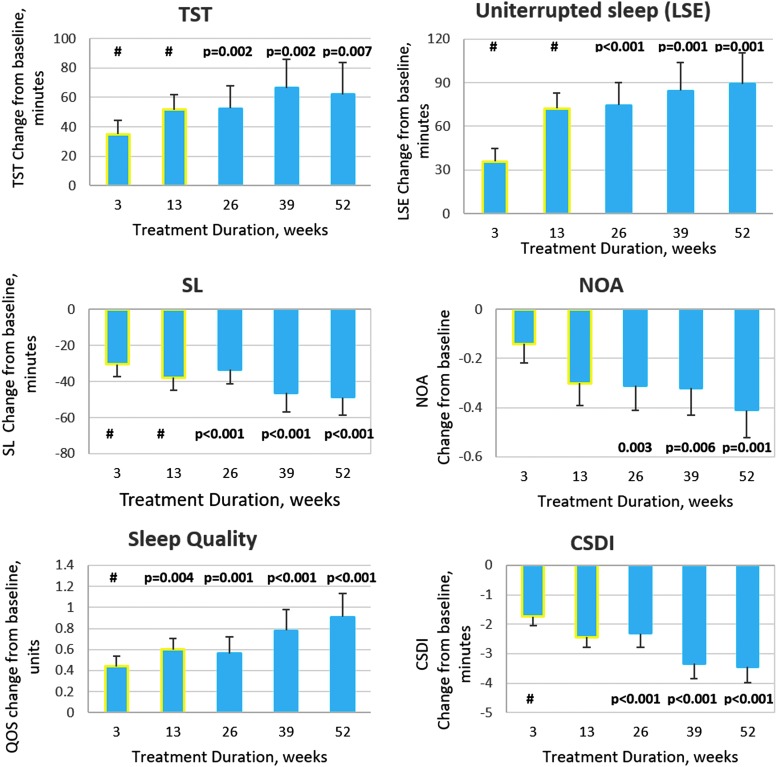
Effects of continuous PedPRM treatment (52 weeks) on child sleep. SND-reported change from baseline (end of the single-blind placebo run-in phase) in mean (SE) TST (minutes), SL (minutes), duration of uninterrupted sleep (LSE, minutes), NOA, quality of sleep, and CSDI in the PedPRM-assigned group during the 52 weeks of continuous treatment (*n* = 41). For completion of the picture, data from the 13-week, double-blind phase for this group (Gringras et al. 2017) are also depicted and marked with *yellow outline*. ^#^ represents significant differences over placebo in the double-blind phase (MMRM analysis; Gringras et al. 2017). CSDI, Composite Sleep Disturbance Index; LSE, longest sleep episode; NOA, number of awakenings; PedPRM, pediatric prolonged-release melatonin; SL, sleep latency; SND, Sleep and Nap Diary; TST, total sleep time.

The mean (SE) changes from baseline in SND-reported sleep measures (TST, SL, LSE, QOS, and NOA) during 52 weeks of continuous PedPRM treatment (subjects who had been assigned to PedPRM in the double-blind phase; *n* = 41) are presented in Figure 2. For ease of interpretation, data on this group from the double-blind phase (Gringras et al. 2017) are also depicted (yellow outline). As can be seen, improvements in TST (increase), SL (decrease), and LSE (increase) observed in the double-blind phase were maintained or enhanced throughout the follow-up (Fig. 2). After 52 weeks of continuous PedPRM treatment, children slept on average (mean [SE]) 62.08 (21.5) minutes longer (*p* = 0.007); duration of uninterrupted sleep (LSE) increased by 89.1 (25.5) minutes (*p* = 0.001); SL shortened by 48.6 (10.21) minutes (*p* < 0.001); nightly awakenings NOA decreased by 0.41 (0.12; >50%; *p* = 0.001); and quality of sleep improved significantly (*p* < 0.001) compared with baseline values (Fig. 2).

Subjects originally assigned to placebo also improved with PedPRM treatment. By the end of 39 weeks of follow-up these children slept on average (mean [SE]) 25.6 (17.2) minutes longer (N.S.); duration of uninterrupted sleep (LSE) increased by 67.2 (22.99) minutes (*p* = 0.007); SL shortened by 33.6 (8.3) minutes (*p* < 0.001); nightly awakenings NOA decreased by 0.38 (0.16; *p* = 0.021); and quality of sleep improved significantly (*p* = 0.001) compared with baseline values. Although improvements in this group lagged behind those in the patients who were assigned to PedPRM from start, there were no significant differences between the groups, thus allowing us to combine the groups during the follow-up phase (Table 1).

**Table 1.** Sleep Variables After 13, 26, and 39 Weeks of Open-Label, Pediatric Prolonged-Release Melatonin Treatment of the Combined Population^a^

*Variable*	*13 weeks open label*	*26 weeks open label*	*39 weeks open label*
n^b^	*91*	*79*	*72*
TST (minutes)			
Estimated change from baseline^c^ (SE)	37.01 (10.26)	40.75 (12.34)	44.35 (13.94)
*p*	0.001	0.001	0.002
SL (minutes)			
Estimated change from baseline (SE)	−28.39 (5.68)	−41.9 (6.34)	−41.36 (6.64)
*p*	<0.001	<0.001	<0.001
NO^a^			
Estimated change from baseline (SE)	−0.35 (0.08)	−0.38 (0.09)	−0.39 (0.1)
*p*	<0.001	<0.001	<0.001
Longest sleep duration (minutes)			
Estimated change from baseline (SE)	64.21 (12.58)	76.0 (15.5)	78.63 (17.18)
*p*	<0.001	<0.001	<0.001
Quality of sleep			
Estimated change from baseline (SE)	0.53 (0.10)	0.67 (0.12)	0.72 (0.14)
*p*	<0.001	<0.001	<0.001
Sleep disturbance (CSDI)			
Estimated change from baseline (SE)	−2.46 (0.330)	−3.12 (0.34)	−3.27 (0.35)
*p*	<0.001	<0.001	<0.001

^a^ Patients in PedPRM-randomized group had altogether 52 weeks and those in the placebo group had 39 weeks of continuous PedPRM treatment by the end of the 39-week, open-label phase.

^b^ All patients who provided SND data.

^c^ Baseline—2-week, single-blind placebo run-in before randomization.

CSDI, Composite Sleep Disturbance Index; NOA, number of awakenings; PedPRM, pediatric prolonged-release melatonin; SND, Sleep and Nap Diary; TST, total sleep time.

In the combined groups (Table 1), improvement was maintained or enhanced over the 39-week follow-up after attaining the optimal dose, with significant improvements over baseline in TST, SL, uninterrupted sleep duration, NOA, and quality of sleep. Fifty-five (76%) of the 72 patients who completed the 39-week follow-up and who provided SND data achieved an overall improvement of 1 hour or more in TST, SL, or both over baseline and, according to the criteria used in the study, did not require further dose escalation. The average daily dose after 1 year of treatment in patients who satisfied this criterion was 5.3 mg (29% of these subjects used 2 mg/day, 47% used 5 mg/day, and 24% used 10 mg/day). Seventeen subjects (24%) had partial (6 of 17) or no measurable (11 of 17) response compared with baseline even at the highest (10 mg) dose. MMRM analysis, including age, co-medication, diagnosis, or symptom severity, has not shown any particular trait for this group. Diagnosis (ASD with/without ADHD or SMS) and co-medication (e.g., stimulants) did not affect PedPRM efficacy outcomes (data not shown).

For the ASD-only subpopulation mean (SE) improvement in TST in the whole group by the end of 39 weeks of follow-up was 44.5 (14.5) minutes (*p* = 0.002) and SL decreased by 42.55 (6.88) minutes (*p* < 0.001), very similar to values observed in the total study population (Table 1).

The sleep variables at each time-point were compared with those at baseline to show that the treatment continued to be efficacious. While it seems that the effect of the intervention improved over time, this improvement could be explained by the dose modification, at least in part (Fig. 2; Table 1).

**FIG. 3.**
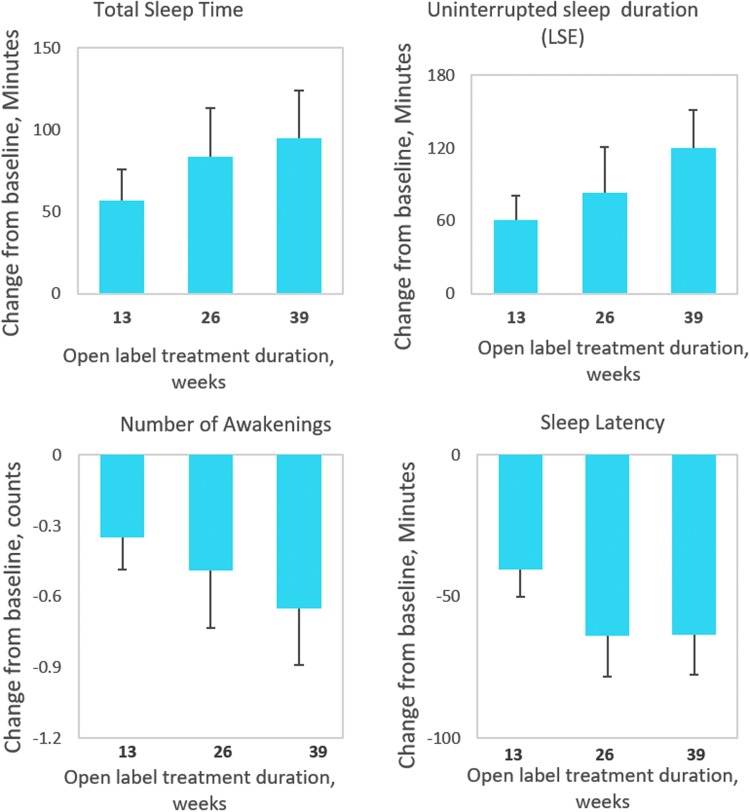
Sustained response to 2 mg PedPRM treatment over the follow-up phase (39 weeks). Change from baseline in mean (SE) SND-reported TST (minutes), SL (minutes), duration of uninterrupted sleep (minutes), and NOA during the 39-week, open-label follow-up in patients treated with 2 mg PedPRM throughout the observation period (*n* = 16). PedPRM, pediatric prolonged-release melatonin; SND, Sleep and Nap Diary.

We explicitly looked at the group treated with 2 mg PedPRM without dose escalation (Fig. 3). Findings of 2 mg treatment give a clinically important information: (a) about lack of attrition effects and (b) that there is a substantial group of children responding to lower PedPRM doses. This group comprised 11 subjects who had been randomized to PedPRM and remained on the 2 mg dose (1 participant had a temporary dose escalation to 5 mg at week 3 but was returned to 2 mg after week 13 of the follow-up) and 5 participants who had been randomized to placebo and given 2 mg PedPRM in the 39 weeks of follow-up without dose escalation (2 participants were randomized to placebo, had—due to dose escalation to 5 mg placebo at week 3—received temporarily 5 mg PedPRM dose during the first 13 weeks of follow-up but their dose was reduced to 2 mg afterward). As can be seen in Figure 3, there was no evidence of decreased efficacy of PedPRM over the 52 weeks. On the contrary, patients staying on the 2 mg PedPRM dose had the same or greater benefits over time without dose escalation (Fig. 3).

The average optimal dose for younger children in the cohort was 5.6 mg, while for the adolescents the mean dose was 8.3 mg, but the dose range was the same across all age groups. The response (change in TST and/or SL) did not differ significantly between the age groups (Table 2).

**Table 2.** Dose and Response by Age After 1 Year of Continuous Study Medication in Children with Autism Spectrum Disorder

*Age, years*	n	*Mean dose (range), mg*	*Mean (SE) change in TST, minutes*	*Mean (SE) change in SL, minutes*
2–7	28	5.64 (2–10)	52.36 (26.52)	−48.66 (8.87)
8–11	20	5.20 (2–10)	45.12 (26.46)	−41.89 (12.31)
≥12	24	8.33 (2–10)	34.37 (18.58)	−32.40 (13.80)

SL, sleep latency; TST, total sleep time.

#### Composite Sleep Disturbance Index

The mean (SEM) change from baseline in total CSDI score in patients treated continuously with PedPRM for 52 weeks is presented in Figure 2. By the end of the 52-week follow-up, mean (SE) total CSDI score improved (decreased) −3.45 (0.53) units from baseline on average (*p* < 0.001). Similar results were obtained with the whole group regardless of randomization history (Table 1).

#### Caregivers' outcomes

By the end of the follow-up, caregivers of children who had been randomized to PedPRM and treated continuously with PedPRM for 52 weeks had significant improvements in sleep quality (mean [SE] change from baseline in PSQI score *−*2.20 [0.517] units; *p* < 0.001), quality of life (mean [SE] change from baseline in WHO-5 2.41 [0.836] units; *p* = 0.006), and CSDI-assessed satisfaction of their child's sleep patterns (1.95 [0.218]; *p* < 0.001).

**FIG. 4.**
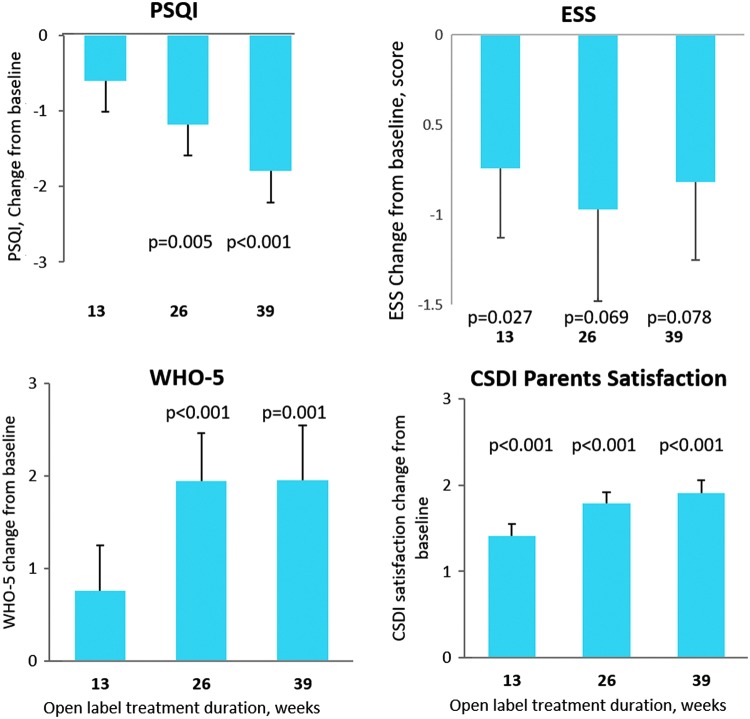
Effects of continuous PedPRM treatment (52 weeks) of the children on their caregivers. Change from baseline in the combined patient groups in mean (SE) caregivers' sleep quality (PSQI), quality of life (WHO-5), ESS, and CSDI-recorded parents' satisfaction of the child's sleep during the 39-week follow-up (*n* = 78). CSDI, Composite Sleep Disturbance Index; ESS, Epworth Sleepiness Scale; PedPRM, pediatric prolonged-release melatonin; PSQI, Pittsburgh Sleep Quality Index.

Changes from baseline in mean PSQI score for the caregivers of the combined groups during the 39-week follow-up are shown in Figure 4. There was a gradual improvement in PSQI over time. By the end of the follow-up mean (SE) PSQI score of the caregivers was lower (improved) by 1.79 (0.42) units compared with baseline (*p* < 0.001). Forty-nine percent of caregivers attained complete remission of insomnia (PSQI score <6; Buysse et al. 1989).

In addition, caregivers' quality of life improved significantly with PedPRM treatment. By the end of 39 weeks of follow-up the mean (SE) WHO-5 increased (improved) by 1.96 (0.59) units compared with baseline (*p* = 0.001; Fig. 4). Approximately 49% (38/77) of caregivers experienced a clinically relevant improvement of 10% or more over the baseline quality-of-life score at week 39 of the follow-up.

Importantly, parents' satisfaction of their child's sleep (CSDI) increased significantly, and by the end of the 39 weeks of follow-up it was improved by mean (SE) 1.9 (0.15) units on a scale of 1–5 (*p* < 0.001; Fig. 4).

Caregivers' daytime sleepiness (ESS) improved significantly after 13 weeks of follow-up compared with baseline with a trend (*p* < 0.1) to improve after 26 and 39 weeks of follow-up (Fig. 4). Thirty-three percent of parents had a relevant reduction of 25% or more on the ESS by the end of follow-up.

### Safety

#### Serious adverse events

Three adverse events were considered “serious” according to standard regulatory definitions, during weeks 13–52 of the study (the first 39 weeks of follow-up). These included (one case each) aggression, oppositional defiant disorder, and constipation. All of these events were considered “not related” to the study medication by review of the physician investigator, and none led to study drug discontinuation.

#### Treatment-emergent adverse events

Out of the 95 patients in the follow-up, 74 patients (77.9%) reported a total of 333 treatment-emergent adverse events (TEAEs) during weeks 13–52 of the study—the first 39 weeks of follow-up (Table 3). Of these TEAEs 26 events in 17 patients were considered treatment-related by review of the physician investigator: 3 of those in 3 subjects (malaise, headache, and insomnia [one case each]) led to study drug discontinuation. There was one patient who temporarily discontinued (interrupted treatment) due to a TEAE (sinusitis) and returned to the study afterward.

**Table 3.** Treatment-Emergent and Treatment-Related Adverse Events

*TEAE*	*Patients*	*% of completers (*n*=95)*	*Events*	*Treatment related TEAEs Patients/%/events*
At least 1 TEAE	74	77.9%	333	17/17.9%/26
Fatigue	18	18.9%	20	5/5.3%/5
Vomiting	17	17.9%	25	
Somnolence	16	16.8%	19	2/2.1%/2
Cough	13	13.7%	20	
Mood swings	13	13.7%	13	3/3.2%/3
Upper respiratory tract infection	10	10.5%	16	
Headache	8	8.4%	8	1/1.1%/1
Rash	8	8.4%	8	1/1.1%/1
Dyspnea	7	7.4%	7	
Constipation	6	6.3%	9	
Nausea	6	6.3%	7	
Pyrexia	6	6.3%	7	
Rhinorrhea	5	5.3%	5	
Aggression	5	5.3%	5	2/2.1%/2
Agitation	5	5.3%	6	1/1.1%/1
Gastroenteritis	4	4.2%	4	
Viral respiratory tract infection	4	4.2%	4	
Asthma	4	4.2%	4	
Hangover	4	4.2%	4	2/2.1%/2
Ear infection	3	3.2%	5	
Influenza	3	3.2%	5	
Lower respiratory tract infection	3	3.2%	4	
Otitis media	3	3.2%	4	
Dizziness	3	3.2%	4	
Seizure	3	3.2%	3	
Tremor	3	3.2%	3	
Dental caries	3	3.2%	3	
Weight increase	3	3.2%	3	
Sinusitis	3	3.2%	3	2/2.1%/2
Irritability	2	2.1%	2	2/2.1%/2
Somnambulism	2	2.1%	2	1/1.1%/1
Psychomotor hyperactivity	2	2.1%	3	1/1.1%/1
Pruritus	2	2.1%	3	1/1.1%/1
Delayed sleep phase	1	1.1%	1	1/1.1%/1
Contusion	1	1.1%	1	1/1.1%/1
Overdose	1	1.1%	1	1/1.1%/1

TEAEs occurring at ≥3% and all TEAEs considered to be treatment-related are depicted.

TEAEs, treatment-emergent adverse events.

The total number of weeks on PedPRM therapy for the 95 patients was 3796 weeks.

The most commonly reported TEAEs were fatigue (18.9%), vomiting (17.9%), somnolence (16.8%), cough (13.7%), mood swings (13.7%), upper respiratory tract infection (10.5%), headache and rash (8.4% each), dyspnea (7.4%), constipation, nausea and pyrexia (6.3% each), rhinorrhea, aggression and agitation (5.3% each). Only in 17.9% of patients the TEAEs were considered by the physician investigator to be definitely, probably, or possibly related to study medication. Of these, the most commonly reported adverse events were fatigue in 5 patients (5.3%); mood swings in 3 patients (3.2%); irritability, aggression, hangover, and somnolence, each in 2 patients (2.1% each). No noticeable changes were found in vital signs at any time-point during the study. Furthermore, there were no differences from baseline in the physical examination. There were small increases in BMI and Z-scores (BMI SD of relative weight adjusted for child age and sex, based on the Center for Disease Control growth charts) in the PedPRM and placebo groups, with no clinically significant difference between the groups. Mean (SE) Z-score increased from baseline by 0.10 (0.049) after 26 weeks (*p* = 0.042), 0.11 (0.063) after 39 weeks (*p* = 0.077), and 0.20 (0.077) after 52 weeks (*p* = 0.013) of study medication as expected for developing children and adolescents and thus not considered related to the treatment. Mean (SE) BMI increased from baseline 0.44 (0.15) after 39 weeks (*p* = 0.005) and 0.69 (0.18; *p* < 0.001) after 52 weeks). No TEAEs related to weight gain or loss were reported in the study.

## Discussion

This study reports 1-year efficacy and safety of a pediatric-appropriate prolonged-release melatonin (PedPRM) in children with ASD (97%) or NGD (3%) with sleep disorders. The beneficial effects of PedPRM on sleep that were demonstrated in the 13-week, double-blind phase over placebo were maintained or augmented throughout the 39 weeks of follow-up by significantly increasing TST, reducing SL, and increasing the duration of uninterrupted sleep (sleep maintenance) compared with baseline. In addition, quality of sleep and NOA improved significantly over the long-term follow-up compared with baseline. The results of SND were corroborated by CSDI, showing that children had reduced sleep disturbances. Altogether 76% of children (3 of 4) had long-term benefit from PedPRM at the optimal dose (2, 5, or 10 mg daily), achieving 60 minutes or more of longer sleep duration and/or shortening of SL.

There were no serious treatment-related adverse effects. Adverse effects were few and generally mild, with fatigue emerging as the main TEAE. Mood swings were in part ascribed to the treatment. Notably, during the double-blind phase, as well as the follow-up, mood swings occurred more in the placebo than PedPRM-assigned group (11 vs. 9 in the double-blind and 8 vs. 5 in the follow-up phase, respectively), and most were not considered related to treatment. Children and adolescents with ASD can have more frequent or more severe mood changes than typically developing teenagers (Arnold et al. 2006), and as this is an open-label study, the mood swings considered treatment related may in fact be due to the underlying disorder.

A clinically described difficulty with some melatonin formulations has been the gradual loss of effect over time (Braam et al. 2010, 2013). Looking at the evolution of efficacy in patients receiving PedPRM without any dose escalation, it appears that they remained responsive throughout the study and no increase in dosage was necessary to avoid attrition of effects. Rather, the response further evolved gradually, and these improvements were maintained throughout the rest of the follow-up.

A limitation in this follow-up study was the open-label design of the study. It is therefore pertinent to ask whether the apparent increase in efficacy with time is a drug-related clinical benefit or that it is linked to spontaneous remission of insomnia. Spontaneous remission of insomnia that might contribute to the overall improvement during the 39 weeks of follow-up in some patients cannot be ruled out. However, taking the known persistence of insomnia in this population (Sivertsen et al. 2012), this is uncommon.

Another limitation in our study was that due to the design of the double-blind phase, most (∼80%) patients in the placebo group were escalated to the 5 mg (placebo) dose and therefore had a starting dose of 5 mg PedPRM in the open-label follow-up (Gringras et al. 2017). It is assumed that if these patients would have started 2 mg PedPRM treatment, a considerable number (up to 40%) would have stayed on the 2 mg dose as was seen in the PedPRM-assigned group.

Because this study treatment of sleep disorder problems in children with ASD was the primary purpose of medical intervention and not to improve core symptoms related to the ASD, the effects of the treatment on ASD severity and comorbid intellectual disability remain to be explored.

Disrupted melatonin secretion in ASD can explain some of the difficulties seen in these patients regarding initiating and maintaining sleep (Melke et al. 2008; Hu et al. 2009; Glickman 2010; Jonsson et al. 2010; Pagan et al. 2011; Tordjman et al. 2012). The enhanced efficacy over time with PedPRM in a number of sleep and daytime variables may perhaps be attributed to improvement in the adjustment of the circadian system to the day–night cycle (Wade et al. 2011; Roth et al. 2015).

Of note, clinicians, parents, and children could decide at any time during the study to discontinue the treatment and end study participation without the necessity to declare any reasons for it. In fact, in most cases, participating clinicians, parents, and children decided to continue PedPRM treatment during the follow-up period, possibly due to ongoing improvement seen even after several months of treatment. Furthermore, benefit to caregivers was also noticed, with improvement in sleep quality (PSQI), daytime sleepiness (ESS), quality of life (WHO-5), and parents' satisfaction of their child's sleep patterns. Disturbed child's sleep has a negative impact on the whole family's health and well-being and impairs their proper employment or further education (Gail Williams et al. 2004; Devnani and Hegde 2015; May et al. 2015). Parents of children with neurodevelopmental disorders who reported that their child had a sleep problem were more likely to have their own sleep disturbed by the child (Robinson and Richdale 2004). The results of this study, showing clinically relevant improvements in caregivers' sleep and quality of life, beyond the beneficial effects on the child, corroborate the importance of improving sleep in the children and adolescents to reduce family distress.

## Conclusions

PedPRM, an easily swallowed formulation of prolonged-release melatonin, is shown to be a safe and effective option for long-term treatment of insomnia in children with ASD or NGD and is also associated with improvement in caregivers' quality of life. Effects are seen rapidly and maintained long term, with no sign of decreased efficacy throughout the 52 weeks of study. Efficacy was demonstrated in terms of significantly increased TST, reduced SL, longer uninterrupted sleep period, improved quality of sleep, and reduction in mid-sleep awakenings. Parents' sleep quality and quality of life improved. Adverse events on PedPRM treatment included fatigue and mood swings. No unexpected safety issues were reported.

## Clinical Significance

Children with ASD have a disproportionally high prevalence of insomnia compared with typically developing children. Data on effectiveness/efficacy of current treatments for the sleep problem are quite limited, and some medications have problematic, potential side effects. PedPRM, an easily swallowed formulation shown to be efficacious versus placebo, is shown to be an efficacious and safe option for long-term treatment (up to 52 weeks reported here) of children with ASD and NGD, suffering from insomnia, and subsequently improves caregivers' quality of life.
